# Intrapulmonary migration of a Kirschner wire 12 years after clavicular fracture fixation: case report and review of literature

**DOI:** 10.1093/jscr/rjaf377

**Published:** 2025-06-06

**Authors:** Robbe Van Dyck, Georges Decker

**Affiliations:** Department of Thoracic Surgery, Hôpitaux Robert Schuman, 20-30 rue d'Anvers, Luxembourg City L-1130, Luxembourg; Department of Thoracic Surgery, Hôpitaux Robert Schuman, 20-30 rue d'Anvers, Luxembourg City L-1130, Luxembourg

**Keywords:** intrapulmonary migration, Kirschner wire, minimally invasive surgery, video assisted thoracoscopic surgery (VATS), case report

## Abstract

Kirschner wires are still widely used for osteosynthesis in orthopedics and trauma surgery. Breaking of this material and migration into the lung parenchyma is a complication that has been occasionally described. We report a case of a patient who presented at our clinic with left thoracic discomfort and an intermittent non-productive cough. Chest X-ray showed a broken clavicular pin with the distal half inside the left chest. The pin was extracted from the lung parenchyma using a left 3-port video-assisted thoracoscopic approach. A literature review suggests that all intrathoracically migrated material should be removed, because of the risk of further migration and harm to the heart or major broncho-vascular structures. A minimally invasive approach should be considered whenever anatomy, clinical presentation and location of the material allows this.

## Introduction

Kirschner wires (K-wires) are sterilized, sharpened, smooth steel pins. These wires have been widely used for osteosynthesis in orthopedics and trauma surgery but are now more restricted to pediatric and hand surgery [1]. Clear advantages of a K-wire are the relative ease of its insertion and removal, which causes minimal trauma. Should this osteosynthesis material (OM) remain in place for an extended period, breaking, and migration of these wires might occur. Although these events are rare, they have been described various times in the literature, causing major complications or even death [2]. Most frequently these migrations lead into the lung parenchyma rather than the pleural space whereas some reports of migration into major mediastinal blood vessels exist [3]. The physiology of this migration into the thorax remains uncertain. We report a case of migration of a broken K-wire into the left lung long after a left clavicular fracture fixation.

## Case report

**Figure 1:**
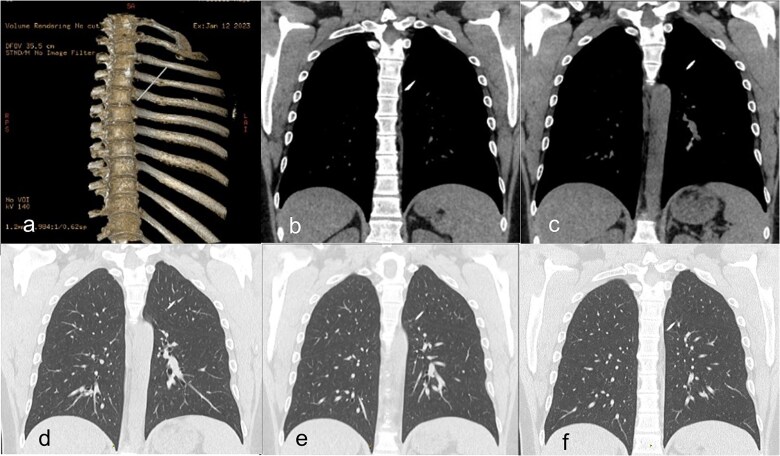
(a) Preoperative CT scan with 3D-reconstruction of skeletal structures and Kirschner wire. (b–f) Distal pin in the left lung transfixing the left upper lobe and fissure into the left lower lobe without pneumothorax.

A 38-year-old right-handed male, active smoker and non-physical worker without sports activities had a car accident in Tunisia. A displaced left mid-clavicular fracture was fixated with a single intramedullary pin. No further information about the accident and initial intervention were available. Twelve years later, the patient presented in our clinic with left thoracic discomfort and an intermittent dry cough. Chest X-ray showed a broken wire with the proximal half still in the clavicle and the distal half inside the left chest. A computed tomography (CT) scan confirmed that the wire had broken at the level of the clavicular fracture, which had consolidated in Z-deformation while the distal half of the pin had migrated into the left lung, transfixing the posterior part of the left upper lobe, passing through the fissure into the apical segment of the left lower lobe. The sharp point of the pin pointed in the direction of the vertebral column directly behind the descending aorta (Fig. 1). The imaging showed no clear inflammatory reaction or abnormalities of the lung parenchyma around the pin.

**Figure 2:**
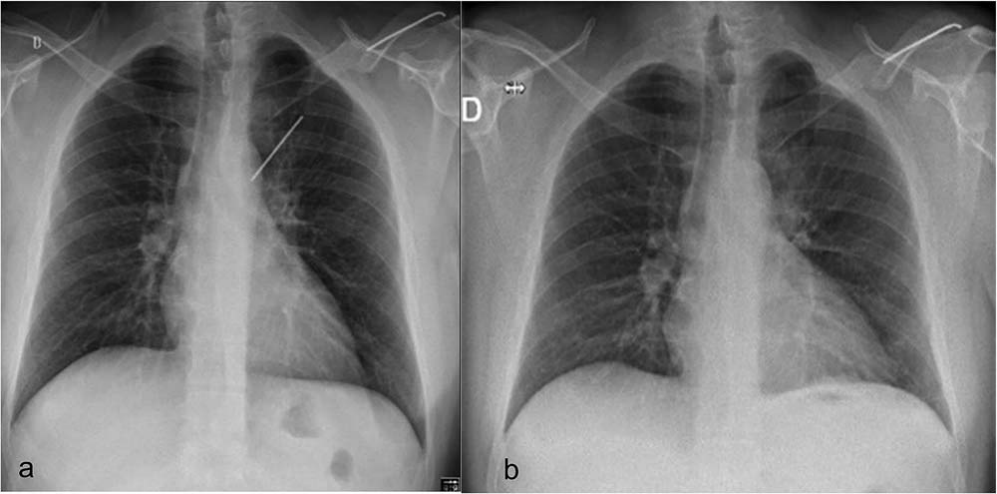
(a) Preoperative chest radiograph with 6.2 cm long K-wire in the left hemithorax. The lateral half of the pin (6.4 cm), located in the distal part of the left clavicle. (b) Postoperative chest radiograph with complete lung expansion after chest tube removal.

Surgical removal of the K-wire was indicated because of the symptoms and the risk of further migration with harm to elements of the bronchial or vascular system. A preoperative chest X-ray confirmed that the position of the pin was unchanged since the CT scan (Fig. 2).

**Figure 3:**
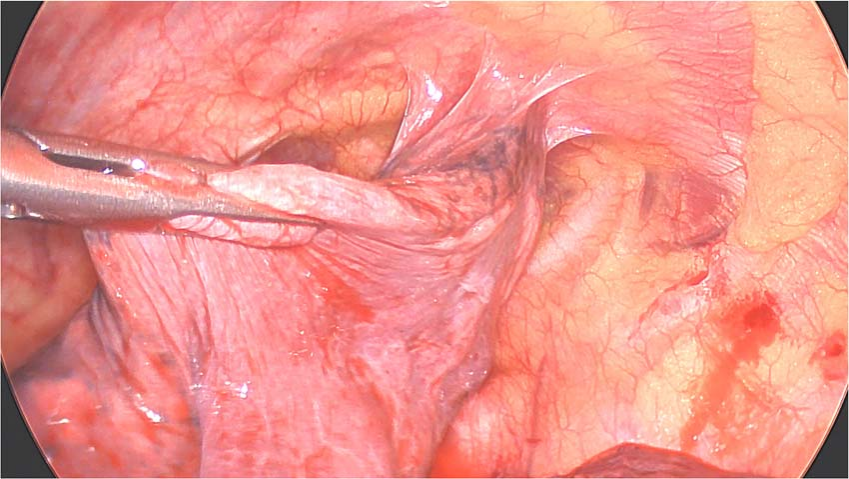
Perioperative views of the migrated K-wire, showing dense apical adhesions of the left upper lobe.

Under general anesthesia with a left double-lumen intubation a left thoracoscopy (VATS) was done using one 11 mm trocar and 2 trocars of 5 mm and a 30° 5 mm camera. We saw a pleural cavity with dense apical adhesions of the left upper lobe against the inferior border of the clavicle (Fig. 3).

**Figure 4:**
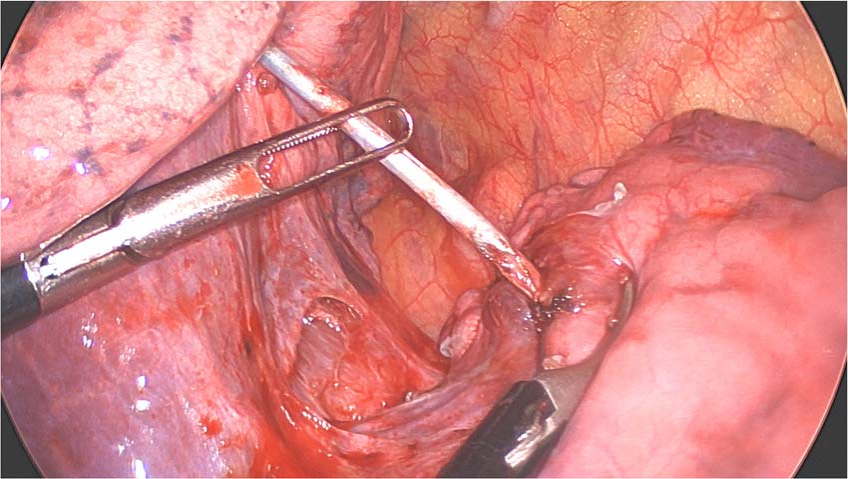
Distal part of K-wire stuck in segment 2 and the proximal part in the apical segment 6.

**Figure 5:**
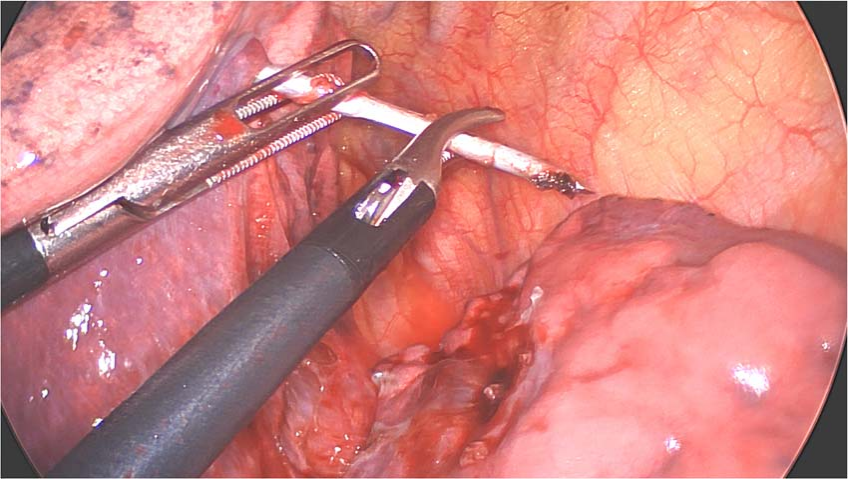
Extraction of the K-wire by pushing the parenchyma towards both extremities.

A clear minor pleural effusion and translucent scissural and para-aortic adhesions were visible. The adhesions of the pulmonary apex were released but no abnormality of the parietal pleura nor bone was visible. As the lung then collapsed, the pin became spontaneously visible protruding in the main fissure. As expected, the proximal part was in segment 2 and the distal half inside the apical segment of the lower lobe (S6) but it did not protrude outside of the parenchyma on either side (Fig. 4). The K-wire was easily removed by gently pushing back the parenchyma towards both extremities (Fig. 5). The pin seemed to be surrounded by a fine membrane that was coagulated at the visceral pleural entry point.

**Figure 6:**
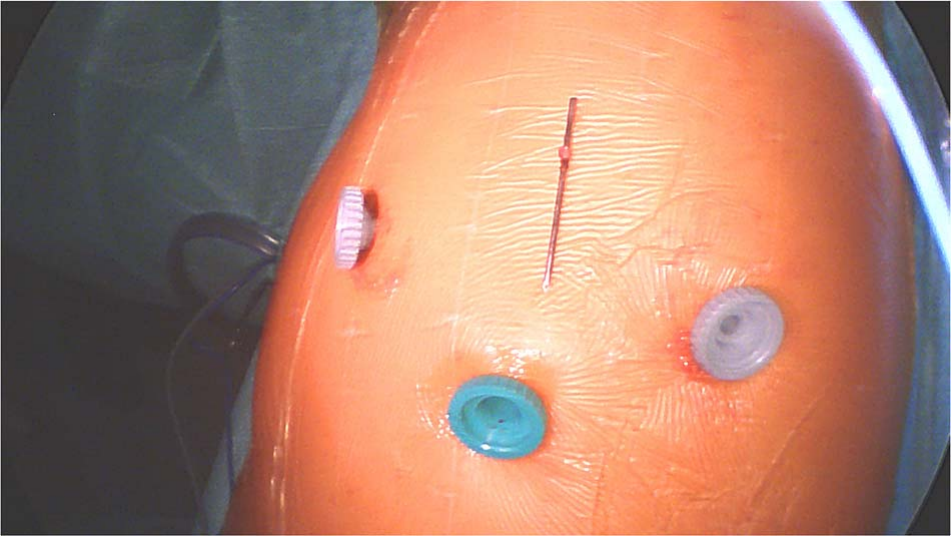
Overview of extracted K-wire and thoracoscopic ports.

There was no spontaneous bleeding. For pneumostasis a single 3/0 PDS suture was placed on the visceral pleural exit site respectively the entry site of both lobes with subsequent negative air leak test. The chest was drained with a 24F drain through the lower trocar port wound (Fig. 6). After intercostal blocks with local anesthetics (Levobupivacaine 10 ml) the lung was insufflated under visual control. The total duration of procedure was 64 minutes and estimated total blood loss was <10 ml. The drain was removed on the first postoperative day (Fig. 2b) and the patient discharged on the second day. At 3-week follow-up he had no residual pulmonary symptoms, took no analgesic medication and had a normal X-ray. The broken proximal half of the wire was left in place. The patient gave his explicit consent for the publication of his case.

## Discussion

Although multiple single cases of intrapulmonary migration have been reported in recent literature, the prevalence of K-wire migration remains unclear. A recent systematic literature review compiled 36 individual cases describing migration of OM into the lung parenchyma [4]. They examined the possible mechanisms and pathophysiology of such migrations. The reported origin of OM was the clavicle in 33%, as in our case report. In 92% of all available cases the material had migrated intact, while in our case, it was broken and only the distal fragment had migrated. In 78% minimal or no clinical repercussions of the migration were mentioned, while some cases of pneumothorax (8%) and hemothorax (14%) were reported.

Although no significant statistical difference could be shown, the mean duration of hospitalization was shorter for thoracoscopic removals (3.2 days) compared to thoracotomy cases (6.2 days) [4]. Due to the minimally invasive approach used, our patient was discharged on postoperative Day 2.

Multiple possible mechanisms for such migrations were raised such as the influence of regional bone resorption, gravity, capillary action but also surgical errors with insufficient measures taken to secure the fixating devices (e.g. by bending its end). Respiratory movements and negative intrapleural pressure combined with gravitational force could have a major impact. No conclusions based on the patient’s profile, age, race, or gender, could be made because of the limited medical information provided in most case reports.

Similarly to our case report, Cameliere *et al*. [5] described apical lung adhesions playing a potential role. These pleural adhesions could be caused by traumas, major surgery or previous infections. However, such adhesions could also be secondary to the migration itself and independently of their cause, they could possibly facilitate and even guide a linear migration straight into the lung parenchyma.

The migrated wires were retrieved by thoracotomy in 23 cases (64%) and by VATS in nine cases (25%). Other ways of retrieval included local shoulder incisions, laparotomy or sternotomy. If required, a conversion from VATS to thoracotomy or sternotomy could always safely be performed [6, 7]. Removal of all migrated OM should be recommended to avoid further migration towards vital intrathoracic structures, whenever possible by minimally invasive approaches [4].
